# Allele frequencies and genotype distribution of three metformin transporter polymorphisms in Mexican population and their application in pharmacogenomics of type 2 diabetes

**DOI:** 10.3389/fphar.2024.1466394

**Published:** 2024-11-01

**Authors:** Oscar I. Chávez-Arreola, Brissia Lazalde, Marisol López-López, Alberto Ortega-Vázquez, Quitzia L. Torres-Salazar

**Affiliations:** ^1^ Biomedical Research Unit, Mexican Social Security Institute, Durango, Mexico; ^2^ Juarez University of the State of Durango, Durango, Mexico; ^3^ Department of Biological Systems, Metropolitan Autonomous University-Xochimilco, México City, Mexico

**Keywords:** metformin, transporters, MATE1, MATE2, Oct2, genotyping

## Abstract

**Background:**

Metformin is the first-line antidiabetic therapy for type 2 diabetes in Mexico, despite recent recommendations highlighting alternatives like GLP-1 receptor agonists for individuals with obesity. Metformin elimination is reliant on liver and kidney function, and variants in transport proteins such as Multidrug and Toxin Extrusion Protein 1 (MATE1), MATE2, and Organic Cation Transporter 2 (OCT2) can influence its pharmacokinetics. Understanding these variants’ frequencies in the Mexican population is crucial for tailoring personalized treatment strategies.

**Objective:**

This study aimed to determine the genotypic and allelic frequencies of key variants in metformin transporters within a Mexican population, addressing the interindividual variability in drug response.

**Methodology:**

Genetic analysis was conducted on 101 healthy, unrelated Mexican subjects who were genotyped for the MATE1, MATE2, and OCT2 variants using allele-specific real-time PCR assays.

**Results:**

The allele frequencies were 0.07 for OCT2, 0.23 for MATE1, and 0.67 for MATE2. The g.–66T→C variant was found only in wild-type and heterozygous forms. Comparative analysis indicated significant differences in allele frequencies between this Mexican population and other ethnic groups, highlighting potential implications for metformin efficacy and safety.

**Conclusion:**

This study provides crucial insights into the genetic variability of metformin transporter genes in a Mexican population, offering a foundation for personalized therapeutic approaches in type 2 diabetes management.

## Introduction

As a first-line therapy for the treatment of type 2 diabetes in countries such as México, the antidiabetic drug metformin is frequently prescribed, both as monotherapy and in combination therapy (Alexander et al., 2008). Its use should be consider in adults at high risk of type 2 diabetes, especially those aged 25–59 years with BMI ≥35 kg/m2, higher plasma glucose (≥110 mg/dL) and higher A1C (e.g., ≥6.0%) and in individuals with prior gestational diabetes mellitus. Although American Diabetes Association in according to its new updates release, Glucagon-like Peptide 1 (GLP-1) receptor agonists demonstrated benefits in adults with type 2 diabetes, with overweight or obesity, in countries such as México, is still a primary treatment (American Diabetes Association Professional Practice Committee, 2024). Within the interindividual variability due to genetic factors, the presence of polymorphisms plays a crucial role. In the case of metformin, differences in response may be influenced by absorption, distribution, and elimination processes, primarily through liver and renal pathways. These processes rely heavily on the transporter function of organic cation transporters (OCTs) and Multidrug and Toxin Extrusion proteins (MATEs) (Masuda et al., 2006a).

MATE transporters play an important role in the renal and biliary excretion of endogenous and exogenous organic cationic compounds, including many drugs (Omote et al., 2006). MATE1 and MATE2 facilitate the efflux of several cationic compounds at the apical membrane of epithelial cells. Human MATE1 is highly expressed in the kidney, adrenal gland, liver, skeletal muscle, and several other tissues (Masuda et al., 2006b). OCTs are primarily found in the intestine, liver, and kidney, where they are directly involved in the absorption, distribution, and elimination of organic cationic molecules such as biguanides (metformin), antivirals, and proton-pump inhibitors. These transporters also handle endogenous substrates, for example, neurotransmitters and endocrine molecules (Dudley et al., 2000). There is no gender differences between the expression of these genes in humans mainly for kidneys, suprarenal glands, liver or skeletal muscle.

Although the MATE1 polymorphism (g.–66T→C) did not influence metformin disposition, healthy volunteers who were homozygous for the MATE1 variant allele had a greater glucose lowering response to metformin. As described, SLC47A1 and SLC47A2 are the genes encoding the MATE1 and MATE2 proteins, respectively (Raj et al., 2017). The reduced-expression promoter polymorphisms of MATE1 would presumably result in reduced transporter expression levels, leading to reduced efflux and correspondingly higher tissue levels of metformin. The higher tissue levels of metformin are predicted to associate with a greater pharmacologic response. Patients homozygous for the MATE1 variant allele had a significantly larger relative change in HbA1c levels (i.e., greater response to metformin) than patients carrying at least one reference MATE1 allele (Stocker et al., 2012a).

OCT2 has been identified in the kidney, brain, placenta, intestine, spleen, lungs, and skin. In the kidney, OCT2 is exclusively located in the basolateral membrane of renal cells, where it is responsible for the initial uptake of metformin, which is then transported by other proteins in the apical membrane for excretion. Both rats and humans have OCT1, which transports a smaller amount of metformin due to its lower affinity for this drug compared to OCT2. Therefore, OCT2 plays a fundamental role in the renal clearance of metformin (Stocker et al., 2012a; Kimura et al., 2005b).

The gene encoding OCT2 is located in segment 26 of the long arm of chromosome 6. OCT2 is a protein of 539 amino acids encoded by the SLC22A2 gene, which consists of 11 exons. Various polymorphisms in SLC22A2 have been described, some of which are located in regions directly involved in transport or molecule recognition (Leabman et al., 2002).

In addition to their effects on pharmacodynamics, these transporters play an essential role in the renal elimination of metformin (Meyer zu Schwabedissen et al., 2010). Specifically, OCT2 is involved in the entry of metformin into renal tubular cells, while MATE1 and MATE2 facilitate its efflux into the urine (König et al., 2011; Sato et al., 2008). Studies have shown that a nonsynonymous variation in OCT2 (rs316019) alters metformin renal clearance in healthy volunteers (Chen et al., 2009; Wang et al., 2008). The rs2289669 polymorphism in MATE1 has been studied concerning the pharmacokinetics of metformin and the reduction of hemoglobin A1c levels in type 2 diabetes patients (Becker et al., 2010). However, there is controversy among authors regarding the association between metformin response and HbA1c levels, likely due to interactions between OCT2 and MATE1 receptors in renal clearance (Christensen et al., 2011).

A common variant, g.−130G→A (>26% allele frequency), in the MATE2 receptor, has been shown to result in a lower response to metformin due to promoter hyperactivity in patients with type 2 diabetes (Choi et al., 2009). Homozygous individuals with this polymorphism exhibit significantly lower metformin levels (Stocker et al., 2012b). Other studies evaluating the SLC22A2 gene 808 G→T variant in the OCT2 receptor observed no significant effects on metformin transport *in vitro* (Leabman et al., 2002), lower renal clearance, and no association with metformin response in type 2 diabetes patients (Christensen et al., 2011; Song et al., 2008).

Metformin reduces hepatic glucose output by decreasing gluconeogenesis, promoting glucose uptake from the bloodstream, increasing insulin sensitivity, and improving insulin action.

Recent studies have highlighted the role of genetic variations in influencing metformin response. A genome-wide association study (GWAS) conducted in the Diabetes Prevention Program (DPP) revealed novel ethnic-specific variants associated with the pharmacokinetics and pharmacodynamics of metformin. Specifically, variants in genes like SLC2A2, which encodes the glucose transporter GLUT2, and ENOSF1 have been implicated in metformin’s glycemic response and clearance. These findings emphasize the importance of further investigating genetic influences on metformin response, particularly in diverse populations, to optimize individualized treatment approaches (Li et al., 2023).

**FIGURE 1:**
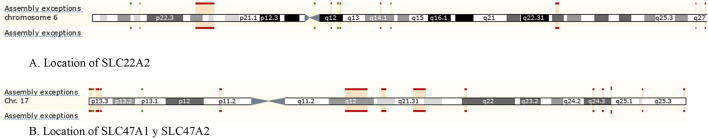
**(A)** Location of SLC22A2. **(B)** Location of SLC47A1 y SLC47A2.

The determination of allelic variants of xenobiotic extrusion genes is not only fundamental for personalized drug prescription but also for understanding allele frequency distribution in different populations (Figure 1, 2). This knowledge should be considered when selecting appropriate medication regimens. The frequency of SLC22A2, SLC47A1, and SLC47A2 gene polymorphisms has been determined and studied in various populations worldwide, including Mexican-Americans, but not in populations within Mexico (Table 1).

**TABLE 1:** Characteristic of studied allele variants of the SLC22A2, SLC47A1 and SLC47A2 genes.

Polymorphism	Allele variant	Amino acide change	Location	Position	Enzyme activity	NCBI dbSNP rs
MATE1/SLC471	g.–66T→C	NM_018242.3:c.-66 =	17p11.2	5′UTR	increased	rs2252281
MATE2-K/SLC472	g.–130G→A	NC_000017.10:g.19619998G>A	17p11.2	5′UTR	decreased	rs12943590
OCT2/SLC222	c.808 G→T	A270S	6q25.3	Exon 4	decreased	rs316019

## Materials and methods

101 healthy unrelated volunteers were included in the study. The study was approved by the ethics committee and informed consent was obtained from each subject prior to their study participation. Each individual was included from an invitation to a general population process to take part on this study voluntarily and were screened to verify they were unrelated and non-consanguineous.

### Isolation of deoxyribonucleic acid

Blood samples were extracted into ethylene-diaminetetraacetic acid (EDTA) tubes. The genomic DNA was prepared using blood according to a commercial procedure (Wizard^®^ Genomic DNA purification kit, Promega, Madison, USA). Isolated DNA samples were subsequently stored at −20°C for use in further analyses. DNA yield and purity were determined by measuring absorbance at 260/280 nm.

### PCR method

The g.–66T→C, rs2252281, g.–130G→A, rs12943590 and c.808 G→T, rs316019 polymorphisms were genotyped by Real-Time PCR using allelic discrimination TaqMan assay. Primers and allele specific dual fluorogenic probes labeled with Fam as a reporter and Vic as a quencher were used to determine the allelic variants.

The RealTime PCR reactions were carried out in a 9.375 μL reaction volume containing 5 μL of sample DNA, 2.5 μL of the Universal Master Mix (Applied Biosystems, Foster City, CA), 0.125 μL TaqMan SNP Genotyping Assays (AHT947W, C___2593951_10, c___3111809_20, Applied Biosystems, Foster City, CA) specific for each allele and 1.75 μL DNase-free, sterile-filtered water per reaction. RealTime PCR was performed on a StepOne™ Real Time PCR System (Applied Biosystems, Foster City, CA) and cycling conditions were as follows: 95°C for 20 min, followed by 40 cycles of 95°C for 15s and 60°C for 20s annealing temperature. Hardy–Weinberg equilibrium of allele frequencies at individual loci was assessed by comparing the observed and expected genotype frequencies using the Chi-squared test.

### Data analysis

The allele frequencies were calculated based on genotype frequencies, assuming Hardy-Weinberg equilibrium. Observed and expected genotype frequencies were compared using the Chi-squared test, confirming that the population was in Hardy-Weinberg equilibrium for all markers. Haplotype analysis was performed using SNPstat software, which provided insights into linkage disequilibrium between loci.

## Results

We performed genotyping for the c.808G→T variant of the SLC22A2 gene, the g.–66T→C variant of the SLC47A1 gene, and the g.–130G→A variant of the SLC47A2 gene using Real-Time PCR in 101 healthy Mexican volunteers. The cohort consisted of 58 females and 43 males, with significant differences observed between the sexes in several key parameters. Males exhibited a higher body mass index (BMI) than females (25.93 ± 4.43 vs 23.70 ± 4.31 kg/m^2^, *p* = 0.012) and a larger waist circumference (86.27 ± 12.09 vs 73.56 ± 10.59 cm, *p* < 0.001). Although systolic blood pressure was significantly lower in females, it remained within normal clinical ranges for both groups (Table 2).

**TABLE 2:** Characteristics of the study population.

	Females n = 58	males n = 43	Total n = 101	*p* ^a^
Age (years)	20.5 ± 1.15	21.16 ± 2.43	20.78 ± 1.83	0.072
BMI (kg/m^2^)	23.70 ± 4.31	25.93 ± 4.43	24.65 ± 4.48	0.012
Waist (cm)	73.56 ± 10.59	86.27 ± 12.09	78.98 ± 12.85	0.000
Systolic blood pressure(mmHg)	102.93 ± 14.35	109.76 ± 11.79	105.84 ± 13.69	0.012
Diastolic blood pressure (mmHg)	69.56 ± 8.01	72.32 ± 7.18	70.74 ± 7.75	0.077
Leucocytes (mg/dL)	6.38 ± 1.75	6.45 ± 1.64	6.42 ± 1.70	0.846
Hemoglobin (g/dL)	14.23 ± 3.66	15.75 ± 1.25	14.85 ± 3.01	0.014
Platelets (10^3^ mm^3^)	253.18 ± 70.59	228.17 ± 48.10	243.02 ± 63.37	0.017^b^
Glucose (mg/dL)	81.92 ± 5.58	82.48 ± 7.22	82.15 ± 6.27	0.671
Creatinine (mg/dL)	0.71 ± 0.09	0.87 ± 0.14	0.77 ± 0.14	0.000
Cholesterol Total (mg/dL)	150.57 ± 28.12	152.48 ± 30.13	151.35 ± 28.81	0.751
HDL (mg/dL)	45.05 ± 8.70	42.35 ± 9.28	43.95 ± 8.99	0.150
LDL (mg/dL)	84.89 ± 21.22	83.87 ± 25.87	84.47 ± 23.10	0.832
VLDL (mg/dL)	21.07 ± 13.23	27.02 ± 14.51	23.48 ± 14.00	0.040
Triglycerides (mg/dL)	104.38 ± 67.17	137.28 ± 72.00	117.75 ± 70.69	0.009^b^
ALT (U/L)	24.98 ± 8.30	34.64 ± 20.18	28.90 ± 15.04	0.000^b^
AST (U/L)	24.91 ± 25.45	25.05 ± 8.44	24.97 ± 20.25	0.017^b^
Alkaline Phosphatase (U/L)	74.29 ± 17.76	81.92 ± 19.20	77.39 ± 18.64	0.048
DHL (U/L)	474.12 ± 512.88	426.33 ± 76.43	454.70 ± 397.43	0.291^b^
CK(U/L)	71.94 ± 31.54	157.79 ± 135.77	106.82 ± 98.77	0.000^b^
CK-MB (U/L)	12.19 ± 14.18	12.48 ± 3.21	12.31 ± 11.07	0.899^b^

n = number of individuals BMI, body mass index.

^a^ = Student´s t between male and females.

^b^ = Mann-Whitney U *t*-test between male and females.

The allele frequencies for SLC22A2, SLC47A1, and SLC47A2 were 0.07, 0.23, and 0.67, respectively. The genotype frequencies were found to be in Hardy-Weinberg equilibrium (HWE) across all loci (*p* > 0.05), with the following observed heterozygosity rates: 47% for SLC22A2, 35% for SLC47A1, and 11% for SLC47A2. The total heterozygosity for the three markers combined was 30% (Table 3).

**TABLE 3:** Expected and observed allelic and phenotypic frequencies in the population.

N = 101		Observed	Expected
MATE1 (g.-66T →C, rs2252281)			
Genotypic Frecuencies	TT	54 (54.0%)	59.5 (58.9%)
	TC	47 (47%)	36 (35.7%)
	CC	0 (0.0%)	5.5 (5.4%)
Allele Frecuencies	T	77%	
	C	23%	
MATE2 (g.-130G →A, rs12943590)			
Genotypic Frecuencies	GG	16 (15.8%)	11.1 (11.0%)
	GA	35 (34.7%)	44.8 (44.4%)
	AA	50 (49.5%)	45.1 (44.6%)
Allele Frecuencies	G	33%	
	A	67%	
OCT2 (c.-808G →T, rs316019)			
Genotypic Frecuencies	GG	88 (87.1%)	87.5 (86.6%)
	GT	12 (11.9%)	13 (12.9%)
	TT	1 (1.0%)	0.5 (0.5%)
Allele Frecuencies	G	93%	
	T	7%	

Haplotype analysis was performed using SNPstat software, a web-based tool for the analysis of association studies developed by Xavier Sole et al. (Solé et al., 2006) The results revealed strong linkage disequilibrium among the three loci in the Mexican population, consistent with previous reports in other populations. Specifically, the g.–66T→C variant presented only wild-type and heterozygous individuals, while the c.808G→T variant was the only one in Hardy-Weinberg equilibrium (*p* = 0.48) (Table 4).

**TABLE 4:** Genotype and Haplotype Frequencies of MATE1, MATE2 and OCT2 SNP, and their Makers in 101 Normal Mexican volunteers.

Observed genotype frequency					Expected haplotype frequency					Heterozygocity (%)		Linkage disequilibrium tab			
M1	M2	O2	NO	%	M1	M2	O2	%	Groups	O	E	*D´*	*D´.B*	*LOD*	*r* ^*2*^
CC	AA	TT	51	8.4	T	A	G	42.6	M1	47	35.7				
CC	AG	TG	47	7.8	T	G	G	27.7	M2	34.7	44.8				
CC	GG	GG	104	17.1	C	A	G	18.4	O2	11.9	12.9				
TC	AG	TG	94	15.5	T	A	T	5.3	M1+M2	40.9	40.3	0.45	0.034–1	0.013	0.174
TC	GG	GG	151	25	C	G	G	4.4	M2+O2	23.3	28.9	0.59	0.013–1	0.1095	0.1126
CC	AG	TT	36	6	T	G	T	1.1	O2+M1	29.45	24.3	0.81	0.13–1	0.083	0.1217
CC	AA	TG	62	10.23	C	A	T	0.05	M1+M2+O2	31.2	31.1				
TT	GG	GG	158	26.07	C	G	T	0							
Total				116.1				100							

M1, M2, and O2 are MATE1, MATE2 SNP, and OCT2 polymorphisms, respectively. O is observed heterozygosity calculated from the number of heterozygous genotypes/total number of genotypes surveyed. E is expected heterozygosity calculated from allele/haplotype frequencies supposing Hardy–Weinberg equilibrium (1-∑iPi 2 X 100 where Pi is the frequency of the *i*th allele/haplotype). D´, D´.B, LOD, and r2 are the value of D′ between the two loci, 95% confidence upper and lower bounds on D0, the log of the likelihood odds ratio, and the correlation coefficient between the two loci.

A) Expected haplotypes estimated by SNPStat, software based on genotype frequencies (refer to Results, paragraph 4).

B) There was no significant difference between the observed genotype frequencies with those expected under Hardy–Weinberg equilibrium.

C) There were significant differences between the observed multilocus heterozygosities and expected heterozygosities. Only the numbers of expected genotypes which were ≥9, were used for M1+M2 and O2+M1 comparisons (χ2 test, χ2 > χ20:95, 3 degrees of freedom). Only the numbers of expected genotypes, which were ≥5, were used for M2+O2 and M1+M2+O2 comparisons (χ2 test, χ2 > χ20.99, 4 degrees of freedom).

## Discussion

Our study presents the first analysis of key metformin transporter polymorphisms in a Mexican population, revealing allele frequencies that differ significantly from those reported in other ethnic groups. For instance, the g.–66T→C allele frequency in our cohort contrasts with those found in Mexican-Americans, African-Americans, and Asian-Americans, suggesting that these genetic differences could influence metformin’s pharmacokinetics and pharmacodynamics in this population.

Except for the MATE-2 polymorphism g.-130G→A, the analyzed polymorphisms presented similar frequencies to those found in reference studies. Significant differences were observed between our g.–66T→C allele frequencies and those reported for Mexican-Americans [C = 0.289/T = 0.711, χ2 = 0.01, 1 degree of freedom (DF)], African-Americans (C = 0.445/T = 0.555, χ2 = 0.10, 1 DF), European-Americans (C = 0.321/T = 0.679, χ2 = 0.02, 1 DF), and Chinese-Americans (C = 0.231/T = 0.769, χ2 = 0.00, 1 DF). Additionally, significant differences occurred between our g.–130G→A allele frequencies and those of Mexican-Americans (A = 0.341/G = 0.659, χ2 = 0.22, 1 DF), African-Americans (A = 0.277/G = 0.723, χ2 = 0.31, 1 DF), European-Americans (A = 0.262/G = 0.738, χ2 = 0.33, 1 DF), Asian-Americans (A = 0.485/G = 0.515, χ2 = 0.07, 1 DF), and Koreans (A = 0.441/G = 0.559, χ2 = 0.11, 1 DF). Furthermore, significant differences were found between our c.808G→T allele frequencies and those of Mexican-Americans (T = 0.015/G = 0.985, χ2 = 0.04, 1 DF), Asian-Americans (T = 0.086/G = 0.914, χ2 = 0.00, 1 DF), Pacific Islanders (T = 0.071/G = 0.929, χ2 = 0.00, 1 DF), African-Americans (T = 0.011/G = 0.989, χ2 = 0.04, 1 DF), Japanese (T = 0.0168/G = 0.983, χ2 = 0.03, 1 DF), and Koreans (T = 0.014/G = 0.986, χ2 = 0.04, 1 DF).

Although many new drugs have been developed for type 2 diabetes mellitus, metformin remains widely accepted as a first-line therapy due to its low incidence of microvascular and macrovascular events and its beneficial effects on plasma lipids and body weight. There is no validated genetic predictor for metformin response or pharmacokinetics, and epistatic mechanisms (gene-gene interactions) may be important (Becker et al., 2010). Investigating genetic variants in specific patient populations, as well as gene-gene and gene-environment interactions, may elucidate important polymorphisms. Furthermore, advances in sequencing platforms, whole-exome or genome sequencing will facilitate the investigation of rare variants, insertions, deletions, and other genetic variants that may play a significant role in metformin pharmacokinetics and response (Lee and Kwon, 2004)

Given the focus of this study on genetic variants related to metformin transport in healthy individuals, it is important to highlight the necessity for further validation in patients actively receiving metformin therapy. Studying individuals not currently on metformin allowed for the identification of baseline genetic polymorphisms associated with drug transport and elimination mechanisms. However, the functional impact of these variants on metformin pharmacokinetics and pharmacodynamics warrants investigation in clinical settings where the drug is administered. Future studies should include participants undergoing metformin treatment to directly assess the influence of identified polymorphisms in SLC22A1, SLC47A1, and SLC47A2 on drug clearance. Additionally, other potential genetic contributors to metformin metabolism must be explored, given the complex nature of drug transport and elimination. This approach will provide a more comprehensive understanding of the genetic factors influencing metformin response, ultimately contributing to the optimization of personalized therapy.

A key limitation of this study is the absence of direct measurements of metformin elimination in participants actively receiving the drug. The primary aim of this research was to identify the frequency of key polymorphisms in genes involved in metformin transport and clearance, such as SLC22A1, SLC47A1, and SLC47A2, within a Mexican and Latin American population, where these frequencies had not been previously characterized. While we did not administer metformin to the participants, the identification of these genetic variants provides a foundational basis for future studies that will evaluate their functional impact on metformin pharmacokinetics in clinical settings. We acknowledge that further validation is necessary, particularly in patients receiving metformin, to confirm the role of these polymorphisms in drug elimination and response. Future investigations should focus on integrating pharmacogenomic data with clinical trials in order to enhance personalized therapy for patients in this population.

## Conclusion

Allele frequencies of the novel g.–66T→C (rs2252281), g.–130G→A (rs12943590), and c.808 G→T (rs316019) polymorphisms in MATE-1, MATE-2, and OCT-2 respectively, were established, indicating an increased frequency in the Mexican population.

Haplotypes were constructed from sets of genotypes, resulting in seven haplotype groups. Haplotype analysis of these three markers among 101 normal Mexican volunteers showed weak linkage disequilibrium between the polymorphisms, as previously reported in other populations. This is likely due to the proximity of the MATE-1 and MATE-2 polymorphisms during chromosomal segregation and recombination in cellular division.

## Data Availability

The data supporting the findings of this study are available to any qualified researcher upon reasonable request. In accordance with the FAIR principles, the dataset has been organized to be findable, accessible, interoperable, and reusable. The metadata and documentation necessary for interpreting the data are provided to ensure that it can be located and utilized by other researchers. Requests for access to the data should be directed to the corresponding author, and will be granted following institutional and ethical guidelines to ensure appropriate use.
